# Clinical and dosimetric considerations for yttrium-90 glass microspheres radioembolization of intrahepatic cholangiocarcinoma, metastatic colorectal carcinoma, and metastatic neuroendocrine carcinoma: recommendations from an international multidisciplinary working group

**DOI:** 10.1007/s00259-025-07229-8

**Published:** 2025-03-28

**Authors:** Marnix Lam, Riad Salem, Beau Toskich, S. Cheenu Kappadath, Carlo Chiesa, Kirk Fowers, Paul Haste, Joseph M. Herman, Edward Kim, Thomas Leung, Siddharth A. Padia, Bruno Sangro, Daniel Y. Sze, Etienne Garin

**Affiliations:** 1 Department of Radiology and Nuclear Medicine, University Medical Center Utrecht, Huispostnummer E01.132, Postbus 85500, 3508 GA Utrecht, The Netherlands; 2 Univ Rennes, INSERM, INRA, Centre de Lutte Contre Le Cancer Eugène Marquis, Institut NUMECAN (Nutrition Metabolisms and Cancer), 35000 Rennes, France; 3 Department of Radiology, Northwestern Feinberg School of Medicine, Chicago, IL USA; 4 Department of Radiation Oncology, Mayo Clinic, Jacksonville, FL USA; 5 Department of Imaging Physics, University of Texas MD Anderson Cancer Center, Houston, TX USA; 6 Department of Nuclear Medicine, Fondazione IRCCS Istituto Nazionale Tumori, Milan, Italy; 7 Boston Scientific Corporation, Marlborough, MA USA; 8 Department of Radiology and Imaging Sciences, Indiana University School of Medicine, Indianapolis, IN USA; 9 Department of Radiation Medicine, Northwell Health, New Hyde Park, NY USA; 10 Department of Interventional Radiology, Mount Sinai, New York City, NY USA; 11 Comprehensive Oncology Centre, Hong Kong Sanatorium and Hospital, Hong Kong, Hong Kong; 12 Department of Radiology, University of California-los Angeles, Los Angeles, CA USA; 13 Liver Unit, Clinica Universidad de Navarra and CIBEREHD, Pamplona, Spain; 14 Department of Radiology, Stanford University School of Medicine, Palo Alto, CA USA; 15 Department of Nuclear Medicine, Cancer Institute Eugene Marquis, Rennes, France

**Keywords:** Radioembolization, Yttrium-90, Dosimetry, Intrahepatic cholangiocarcinoma, Metastatic colorectal cancer, Neuroendocrine

## Abstract

**Purpose:**

The TheraSphere Global Steering Committee reconvened to review clinical data and address knowledge gaps related to treatment and dosimetry in non-HCC indications using Yttrium-90 (^90^Y) glass microspheres.

**Methods:**

A PubMed search was performed. References were reviewed and adjudicated by the Delphi method. Recommendations were graded according to the degree of recommendation and strength of consensus. Dosimetry focused on a mean dose approach, i.e., aiming for an average dose over either single or multicompartment volumes of interests. Committee discussion and consensus focused on optimal patient selection, disease presentation, liver function, tumour type, tumour vascularity, and curative/palliative treatment intent for intrahepatic cholangiocarcinoma (iCCA) and colorectal and neuroendocrine carcinoma liver metastases (mCRC, mNET).

**Results:**

For all indications, single compartment average perfused volume absorbed dose ≥ 400 Gy is recommended for radiation segmentectomy and 150 Gy for radiation lobectomy. Single compartment 120 Gy for uni- and bilobar treatment reflects current clinical practice, which results in variable tumour and normal tissue absorbed doses. Therefore, multicompartment dosimetry is recommended for uni- and bilobar treatment, aiming for maximum 75 Gy to normal tissue and 150–200 Gy (mCRC, mNET), ≥ 205 (iCCA) tumour absorbed doses. These dose thresholds are preliminary and should be used with caution accounting for patient specific characteristics.

**Conclusion:**

Consensus recommendations are provided to guide clinical and dosimetry approaches for ^90^Y glass microsphere radioembolization in iCCA, mCRC and mNET.

Clinical trial number: not applicable.

**Supplementary Information:**

The online version contains supplementary material available at 10.1007/s00259-025-07229-8.

## Introduction

Worldwide implementation of Yttrium-90 (^90^Y) radioembolization for the treatment of primary and metastatic liver cancer has dramatically increased over the past decade. ^90^Y radioembolization is incorporated into the National Comprehensive Cancer Network (NCCN), European Society for Medical Oncology (ESMO), and European Association for Study of the Liver (EASL) guidelines for cholangiocarcinoma (iCCA) [1–3], metastatic colorectal cancer (mCRC) [4, 5], and neuroendocrine cancer (mNET) [6, 7], and is now a key component in the treatment algorithm for liver tumours [8].

Despite the growing adoption of radioembolization in HCC, treatment strategies for non-HCC indications are less mature [9, 10]. As with HCC, there is a need for refinement of dosimetry-based treatment planning. Two dosimetry models based on the Medical Internal Radiation Dose (MIRD) schema, single compartment dosimetry (SCD) and multicompartment dosimetry (MCD), are generally applicable for treatment of primary and secondary liver cancer [11, 12]. Additionally, voxel-based dosimetry generates dose volume histogram curves, identifying the minimum absorbed dose delivered to a percentage of the tumour volume (e.g., D70 = 70%) and tumour dose distribution in addition to tumour absorbed dose (TAD) and normal tissue absorbed dose (NTAD) [13]. ^90^Y glass microspheres (TheraSphere®, Boston Scientific, Cambridge, MA, USA) are approved for treatment of HCC using SCD and uniform absorbed dose to volume of perfused tissue within a fixed dose range [14–16]. Differences in patient and tumour presentation for iCCA, mCRC and mNET require specific recommendations per indication and with regard to individual patient presentation [11, 12, 17, 18].

Radioembolization treatment planning in non-HCC indications should consider treatment goals (curative/palliative), liver function (underlying liver function and prior treatment exposure) and tumour vascularity. Based on intent, most non-HCC treatments can be categorized into the following four categories: 1) ablative radiation segmentectomy; 2) radiation lobectomy as a bridge to surgery; 3) palliative unilobar disease; 4) palliative bilobar disease [13].

These categories have distinct implications for dosimetry-based treatment planning, which will be further discussed.

## Methods

Two to four members of the committee were assigned to each indication to propose dosimetry recommendations based on contemporary literature review. The full 13-member committee, comprised of hepatology, interventional radiology, medical physics, medical oncology, nuclear medicine and radiation oncology reviewed the final draft. Voxel-based dosimetry was outside the scope of this work due to lack of data.

Degree of recommendation (clinical evidence) (Online Resource 1) and strength of consensus (expert panel experience/opinion) (Online Resource 2) are provided [19]. This evidence ranking system was utilized for each recommendation. Consensus was determined based on agreement of committee members during the in-person review panels.

See Online Resource 3 for more method details.

## Results

### Clinical indications

Therapeutic planning and techniques are dependent on treatment intent [13], with general recommendations outlined in Online Resource 4.

### Liver metastases from colorectal cancer (mCRC)

In mCRC, treatment strategies should be based on the possibility of achieving eradication of all metastases and/or a ‘no evidence of disease’ status, either initially, after surgery, or following systemic therapy [4]. Both ablative intent radiation segmentectomy and radiation lobectomy as a bridge to surgery have that aim.

Multiple institutions have reported on radiation segmentectomy, including tumour types such as iCCA, mCRC and mNET [10, 20–23]. Based on published data for HCC, treatments for mCRC have utilized a single compartment perfused volume absorbed dose threshold of ≥ 200 Gy [10, 22, 24]. In retrospective case series with mCRC patients, reported median TAD was 293 Gy (range 163 – 1303 Gy) [21], mean perfused volume absorbed dose was 269 Gy (119 – 477 Gy) [20] and median perfused volume absorbed dose was 416.4 Gy (321.9 – 1,780 Gy) [22]. ORR, time-to-event endpoints (time-to-progression (TTP) and overall survival (OS)), and safety endpoints were in line with HCC data published for radiation segmentectomy and ablation, acknowledging major differences in biology and study endpoints (e.g., RECIST 1.1. vs. mRECIST) [22]. Recently, this paradigm was confirmed in 36 mCRC patients. Complete radiographic (hazard ratio (HR) = 1.29e-16, *p* < 0.001) and metabolic response (HR = 0.38, *p* = 0.038) correlated with prolonged local tumour progression-free survival (LTPFS). LTPFS rate (1-year) for tumours receiving TAD ≥ 400 Gy was 68.6% versus 14.3% for those receiving < 400 Gy (*p* = 0.013). Mean TAD ≥ 400 Gy was an independent predictor of LTPFS by multivariate analysis [25].

Several case series have demonstrated radiation lobectomy utility for colorectal cancer patients [26–28]. In one case series of three patients, the perfused volume absorbed dose was 206–392 Gy administered in the right lobe, resulting in a 25–119% increase of the contralateral future liver remnant (FLR) volume. Treatment was well tolerated. Some mCRC patients successfully underwent resection following radiation lobectomy [28]. Liver function assessment via hepatobiliary scintigraphy demonstrated a decline in the treated volume while the non-treated volume demonstrated an increase in volume and function [27]. Radiation lobectomy demonstrated a low rate of post-hepatectomy liver failure [29]. Most patients were treated with microspheres in week 1.

Dosimetry assessment for normal tissue becomes more important in the palliative setting, e.g., treating lobar or bilobar disease [13, 30], accounting for most mCRC radioembolization cases. Accuracy may be reduced with high numbers of small relatively hypovascular tumours [31], caused by errors during registration of baseline CT/MRI images with SPECT/CT and by preferential flow to normal liver, both aggravated by multiple hypovascular tumours and the use of technetium-99 macroaggregated albumin ([^99m^Tc]TcMAA). For dosimetry and treatment planning, the focus may shift from applying an effective TAD while maintaining safety, to applying a safe NTAD while optimizing TAD. Target lesions (e.g. 1–3 tumours > 3 cm) may be chosen for TAD calculation. Coregistration of pretreatment [^18^F]fluorodeoxyglucose ([^18^F]FDG) with [^99m^Tc]TcMAA SPECT/CT may help lesion segmentation [32, 33]. Similarly, post-therapy ^90^Y PET/CT dosimetry and metabolic response assessment by [^18^F]FDG PET/CT may identify metabolically active disease [17, 34, 35].

A retrospective analysis of 31 patients showed that a TAD of > 139 Gy by ^90^Y PET/CT predicted a three-month metabolic response with the greatest accuracy (89% specificity, 77% sensitivity), whereas a TAD of more than 189 Gy predicted response with 97% specificity and 45% sensitivity. Initial results showed a median NTAD (averaged over the whole liver, i.e. treated and non-treated normal liver) of 63 Gy (range 24–113 Gy) with no unacceptable toxicity observed up to 88 Gy (i.e., most patients treated with microspheres in late week 1 or early week 2). Toxicity was monitored for three months after treatment. Additional research is necessary to understand how ^90^Y PET/CT correlates with pretreatment dose planning using [^99m^Tc]TcMAA SPECT/CT [17].

Retrospective and prospective studies have evaluated radioembolization in the palliative setting, mostly as a second-line or later option in liver-dominant disease [36–38]. Radioembolization in combination with systemic therapy was evaluated in two prospective studies where the safety and toxicity profile matched the individual components [36, 39]. In the EPOCH phase III trial, the combination of radioembolization plus systemic chemotherapy (*n* = 215) in the second-line setting was studied compared with chemotherapy alone (*n* = 213). Primary endpoints, progression-free survival (PFS) and hepatic PFS (hPFS), were met [36]. Patients were treated by single compartment modelling, 120 Gy (± 10%) perfused volume absorbed dose, with no differentiation between TAD and NTAD. EPOCH was the first positive randomized trial for ^90^Y glass microspheres radioembolization, however, a difference in OS was not achieved. Patients were stratified by unilobar/bilobar disease, first-line systemic chemotherapy (FOLFOX/FOLFIRI) and KRAS status. A statistically different time to subsequent treatment was identified, suggesting a possible role in patients desiring a chemotherapy ‘holiday’. In subsequent subgroup analyses which provided a balanced comparison between study arms, two prognostic factors, Eastern Cooperative Oncology Group (ECOG) status and baseline carcinoembryonic antigen (CEA) level, were identified in addition to KRAS status that may aid in patient selection [37].

Two subgroups were identified with different prognoses. Subgroup A included KRAS, any status, and excluded patients with both ECOG 1 and a baseline CEA ≥ 35 ng/mL, while subgroup B further excluded patients with KRAS-mutant status [37]. Prognosis factors were based on EPOCH primary analysis and published literature [40]. Prolongation of PFS and hPFS were seen with subgroups A and B compared with the intention-to-treat (ITT) population. Subgroup B PFS and hPFS were prolonged compared to subgroup A. CEA response (≥ 25% or ≥ 50%) was used to identify responders or early disease progression. The median time-to-deterioration in quality of life (TTDQoL) was identical between the two arms in the ITT population, while the TTDQoL was statistically longer for both subgroups, with subgroup B being longer than subgroup A in the treatment arm (^90^Y glass microspheres plus systemic chemotherapy). The subgroup analysis aids in setting expectations in treatment outcomes based on prognosis. The safety profile followed a similar pattern, where subgroup B tolerated the combination better than subgroup A, and both were better tolerated than the ITT population [37].

Table 1 lists recommendations for mCRC patients.

**Table 1:** mCRC radioembolization recommendations

Treatment Intent	Treatment intent may be palliation, down-staging or bridging to resection, augmenting systemic treatment or definitive radiation 1. In a limited number of eligible patients, radiation segmentectomy or radiation lobectomy are recommended 2. Most patients are treated with palliative intent due to late stage, multifocal disease with large tumour burden. There is no evidence for routine treatment in the first-line setting, however, compelling evidence exists for treatment in the second- or third-line setting. Concurrent systemic treatment should not be permanently discontinued during radioembolization. Temporary chemotherapy dose reductions or pauses should be considered on an individual patient level [36, 41]
Patient Selection	1. Longer median OS in patients with KRAS-wild type, ECOG 0, baseline CEA ≤ 35 ng/mL and absence of extrahepatic disease [37, 42] 2. Maximum acceptable intrahepatic tumour burden is ~ 50%. The higher the tumour burden, the higher the risk for toxicity and/or rapid disease progression (relative contraindication) 3. Extrahepatic disease burden: ≤ 5 pulmonary nodules and either lymph nodes belonging to one region or one additional metastatic site amenable to future definitive treatment [36]. Controlled extrahepatic disease in combination with uncontrolled liver disease may be an argument for treatment. The (controlled) primary tumour may be in situ
Pretreatment Imaging	1. Arterial and portal venous phase contrast-enhanced MRI or CT is recommended within 4 weeks of treatment to avoid unexpected interval progression 2. [^18^F]FDG -PET/CT (may be combined with contrast-enhanced CT (CECT)) may be performed for staging [17, 34]
Dose Calculation and Dosimetry Considerations	1. Radiation segmentectomy: SCD is adequate (≥ 400 Gy with no established upper limit) [10, 20–23, 43]. In HCC, multiple studies have demonstrated higher rates of complete pathologic necrosis are achieved with higher total perfused doses [44–46]. A specific perfused volume absorbed dose has not been established in mCRC, but studies seem to confirm ≥ 400 Gy [22, 25, 45]. MCD and voxel-based dosimetry may become more important in radiation segmentectomy in the future [25] 2. MCD is preferred over SCD to evaluate TAD and NTAD. Data on specific dose thresholds is limited to post ^90^Y PET/CT [17] 3. Radiation lobectomy: SCD may be used at a 150 Gy average absorbed dose limit given the preserved function in the untreated parts of the liver [26, 28, 47] 4. If extensive tumour involvement precludes radiation segmentectomy or lobectomy, activity should be planned to achieve tumouricidal TADs with tolerable NTAD. High TADs have been shown to correlate with treatment response, whereas high NTADs increase the risk for hepatic decompensation 5. SCD reference average absorbed dose to the perfused volume is 120 Gy. The perfused volume may be the whole liver or a fraction thereof, which has proven to be safe and effective. TAD and NTAD may vary considerably between patients. Apply caution in case of poor targeting ([^99m^Tc]TcMAA SPECT/CT) and/or low tumour burden (< 10%), which could lead to higher toxicity and probability of radioembolization-induced liver disease (REILD), particularly in bilobar patients 6. MCD NTAD prediction is typically more accurate than TAD, especially for (multiple) small, and/or heterogeneous tumours [17]. Although evidence is limited and dose threshold should be regarded as preliminary, a minimum TAD of 150 Gy is advised, preferably > 200 Gy, with a maximum NTAD of 75 Gy for single-session whole liver treatment. Proportionally higher NTAD is acceptable for smaller treatment fractions 7. Prescription decisions on perfused volume and TAD and NTAD should be based on treatment intent relative to clinical status, liver function, tumour load, catheterization, targeting, hypervascularity, and previous treatments. Consider dose reductions in case of toxicity from previous systemic treatment or with concomitant systemic treatment, especially radiation-sensitizing treatment 8. Most committee members recommend week 1 (Wednesday/Thursday/Friday) or early week 2 treatment (Monday/Tuesday) to replicate published outcomes, as a specific activity outside the range described may result in different TAD and NTAD thresholds
Treatment Delivery	1. Bilobar disease can be treated using single session bilobar or staged sequential lobar treatment [17, 36, 48], depending on prior systemic chemotherapy, disease burden, potential for interval progression in an untreated lobe, and NTAD. In patients with low tumour burden (< 10% of normal liver replaced by tumour), sequential lobar treatment is recommended if SCD is used. With staged sequential lobar treatment, the lobe with more extensive disease should be treated first. For highly aggressive bilobar disease in a patient with preserved liver function and good [^99m^Tc]TcMAA tumour targeting (i.e., high TAD; low NTAD), single-session bilobar treatment (i.e. two unilobar injections) should be based on MCD 2. The interval between sequential lobar treatments is 4–8 weeks [48]. A shorter interval may decrease the chance of interval progression but may increase cumulative toxicity. A longer interval may increase recovery, but at the potential cost of interval progression (consider repeat imaging). One-time unilobar [^99m^Tc]TcMAA mapping for lung shunt fraction estimation is sufficient for contralateral treatment but cannot be used for dosimetry purposes [49]
Outcome Assessment and Follow-up	1. Contrast-enhanced MRI or CT should be performed every three months following treatment with consideration for subsequent treatment possibilities [36, 48] 2. [^18^F]FDG-PET metabolic response can be observed 4–6 weeks after radioembolization; response at cross-sectional anatomic imaging may take longer (2–3 months) [17, 34] 3. CEA levels should be included in routine follow-up. CEA response (25% or 50% decrease from baseline) indicates the duration and level of treatment response [37] 4. In the palliative setting, caution is warranted with an overly aggressive retreatment approach in patients with stable disease (SD) or partial response (PR). Retreatment (radioembolization, ablation, or systemic therapy) should be considered only for progressive disease (PD)
Strength of Recommendation	B
Degree of Consensus	Strong

### Liver metastases from neuroendocrine tumours (mNET)

Two case studies including mNET patients presented radiation segmentectomy with a target perfused volume absorbed dose of ≥ 190 Gy [10, 22]. The median perfused volume absorbed dose was 416.4 Gy (range 321.9 – 1,780) [22]. Disease control occurred in 93.3% of oligometastatic patients, median TTP was 72.9 months for the treated lesion and median OS was 60.9 months, consistent with the use of ablative dosimetry in selective infusions in other tumour types. No data is available on radiation lobectomy in mNET patients.

Most mNET patients present with bilobar disease in a palliative setting, which necessitates more accurate dosimetry-based treatment planning, balancing efficacy and toxicity. For the broader mNET population, improving quality of life is an important additional goal of radioembolization [50–55].

As mNET patients have prolonged OS relative to other primary and secondary liver cancers, longer follow-up and the impact of radioembolization on liver function becomes important. In patients with low-grade disease (especially patients with grade 1 disease who generally have a relatively long survival), non-functional tumours, limited hepatic tumour-load and bilobar disease, emphasis should focus on a safe NTAD while maintaining a meaningful TAD (particularly important for higher grade disease). To overcome registration and segmentation issues, the use of target lesions (e.g., 1–3 tumours > 3 cm) may be useful for TAD analysis and treatment planning. In patients with multiple hypervascular lesions and good [^99m^Tc]TcMAA targeting, a SPECT-based thresholding technique may be used for tumour segmentation as an alternative for manual segmentation on CT/MRI. Additional measures may include a two-staged approach or multiple (super)selective administrations. Compared with other tumour types, a two-staged approach has a lower risk of interval progression. An MCD approach is preferable since some reported long-term hepatotoxicity in their series using SCD.

In a retrospective review, mNET patients who underwent either lobar or bilobar radioembolization with a mean follow-up of 4.1 years (range, 2.0 – 15.2 years), were evaluated. Mean perfused volume absorbed dose for a lobar treatment was 114 Gy (range, 11 – 240 Gy) and for repeated treatments was markedly high at 254 Gy (range, 109 – 524 Gy) to the right hepatic lobe, and 375 Gy (range, 175 – 762 Gy) to the left hepatic lobe. TAD and NTAD were not taken into account, which limited toxicity analysis [50]. Relative to lobar radioembolization, sequential lobar radioembolization of the whole liver, exhibited higher rates of cirrhosis-like morphology and portal hypertension, however, the majority remained clinically asymptomatic. Significant long-term hepatotoxicity related to radioembolization developed in a small percentage [50]. mNET patients are often treated with a variety of locoregional and systemic treatments, which all contribute to hepatic dysfunction over time.

In a 26-patient study, the institution treatment algorithm included mNET patients following progression on peptide radionuclide receptor therapy (PRRT). The dose–response relationship identified median 170 Gy in responding tumours, median 101 Gy for SD, and median 67 Gy for PD, with 29% overall response rate (ORR) [11]. Tumour response demonstrated a statistically significant association with TAD. The median perfused volume absorbed dose was 120 Gy (range, 30 – 200 Gy). A significant dose-toxicity relationship was not identified, although two patients developed REILD with a total healthy liver dose of 110 Gy and 128 Gy. The geometric mean for responding tumours was 194 Gy (IQR [1236, 291]), 114 Gy (IQR [62, 178]) for non-responding tumours, *p* < 0.001. The minimum recommended TAD was 150 Gy, preferably > 200 Gy.

In a 43-patient study, treatment response was evaluated using retrospective [^99m^Tc]TcMAA pretreatment MCD analysis of patients who had undergone SCD (range 80–150 Gy) treatment [56]. Most patients underwent single session ^90^Y glass microspheres radioembolization (86%). Dosimetry analysis concluded a mean TAD of 196.6 Gy per lesion or 175.0 Gy per patient predicting treatment response, with response in 75% of patients. Median TAD for responders and non-responders was 268.7 Gy (range; 52.7, 1014.6) and 148.1 Gy (range 78.8, 284.6 Gy), respectively. Multivariate analysis of global PFS demonstrated an association of PFS with tumour grade, tumour origin, and TAD. No factors were identified by multivariate analysis for OS. One patient each developed CTCAE ≥ grade 3 hyperbilirubinemia or elevation in aspartate aminotransferase and alanine aminotransferase within six months of radioembolization.

Table 2 lists recommendations for mNET patients.

**Table 2:** mNET radioembolization recommendations

Treatment Intent	1. In eligible patients, radiation segmentectomy or radiation lobectomy are recommended [10, 57] 2. Palliation (i.e., symptom and tumour control) and/or consolidation complementary to systemic treatment is the intent for advanced disease 3. Most patients are treated with palliative intent due to late stage, multifocal disease with large tumour load [50]. Liver function should be preserved to prevent hepatotoxicity that precludes subsequent treatment (locoregional or systemic therapies) 4. Long-term hepatic fibrosis may have a more negative impact than with other tumour types [50] 5. The preference for bland embolization, chemoembolization, or radioembolization remains a subject of scientific debate [10, 50, 58, 59]. Radioembolization typically has better short-term tolerability and durations of response [56]
Patient Selection	1. All grade disease is acceptable, including neuroendocrine carcinoma (NEC) 2. Acceptable intrahepatic (maximum 50–70%; no maximum in case of palliation and preserved liver function) and extrahepatic disease burden (no defined maximum, as long as prognosis is defined by intrahepatic disease). In general, more extrahepatic disease is acceptable (i.e., grade 1–2 NET) compared with other grade NET and/or other tumour types 3. Patients may receive treatment before or after PRRT. Radioembolization demonstrates acceptable tolerability post-PRRT and does not limit subsequent treatment [11, 60, 61]
Pretreatment Imaging	1. Multiple phase contrast-enhanced MRI or CT is recommended within 4–8 weeks of treatment. Depending on tumour grade, a longer interval is acceptable 2. Somatostatin receptor imaging may be performed for staging in somatostatin receptor positive grade 1–2 NET ([^18^F]FDG-PET/CT in grade 3/NEC)
Dose Calculation and Dosimetry Considerations	1. For radiation segmentectomy/lobectomy, it is recommended to use HCC, mCRC, iCCA guidance as a reference due to limited mNET data 2. MCD is preferred over SCD to evaluate TAD and NTAD [11, 62]. Data on specific dose thresholds is limited to a single institution case series and should be further investigated. Routine clinical use can not be recommended at this time 3. SCD reference average absorbed dose to the perfused volume is 120 Gy. The perfused volume may be the whole liver or a fraction thereof, which has proven to be safe and effective [50, 52]. TAD and NTAD may vary considerably between patients. Caution in case of poor targeting ([^99m^Tc]TcMAA SPECT/CT) and/or low tumour burden (< 10%), which could lead to low efficacy and/or high toxicity, respectively 4. MCD NTAD prediction is typically more accurate than TAD, especially for (multiple) small and/or infiltrative tumours. In case of multiple smaller tumours, segmentation may be challenging, and instead can use a count-based isocontour thresholding technique on [^99m^Tc]TcMAA SPECT/CT 5. Optimal tumour response and OS are attained when the TAD is ≥ 200 Gy with a minimum TAD ≥ 150 Gy (i.e., survival benefit threshold). Early studies showed a median whole liver NTAD of 54 Gy (range 11–128 Gy) with safe maximum administration ≤ 106 Gy [11]. A maximum NTAD of 75 Gy for single-session whole liver treatment is recommended to ensure safety. Proportionally higher NTAD is acceptable for smaller treatment fractions 6. Timing of radioembolization and extent of prior liver treatments should be considered. Evidence of long-term radioembolization effects is sparse and current studies lack clinical and dosimetric parameters [50] 7. In lower grade disease, emphasis is on safety (NTAD); in higher grade disease, emphasis is on efficacy (TAD)
Treatment Delivery	1. The interval between sequential lobar treatments ranges from 3–6 months. Interval progression in the untreated lobe is uncommon (except for grade 3/NEC); longer intervals may decrease the risk of liver decompensation. In cases of palliation, a shorter interval may be preferred 2. Bilobar disease can be treated using single-session bilobar or staged sequential lobar treatment. In general, staged sequential lobar treatment is preferred in low-grade NET to avoid potential long-term toxicity. In case of staged sequential lobar treatment, the lobe with more extensive disease should be treated first. For highly aggressive (i.e., grade 3/NEC) bilobar disease in a patient with preserved liver function and with [^99m^Tc]TcMAA tumour targeting (i.e., high TAD; low NTAD), single-session bilobar treatment (i.e., two unilobar injections) should be based on MCD
Outcome Assessment and Follow-up	1. Contrast-enhanced MRI or CT should be performed every 3–6 months following treatment 2. Late responses are common and may take up to 4–9 months [63] 3. In the palliative intent setting, caution is warranted with an overly aggressive retreatment approach in patients with SD or PR. Retreatment (radioembolization, embolization, or systemic therapy) should be considered only in the setting of PD
Strength of Recommendation	B
Degree of Consensus	Strong

### Intrahepatic cholangiocarcinoma (iCCA)

Imaging features of iCCA can vary greatly (e.g., hypervascularity, areas of necrosis, desmoplastic centers) which can impact OS [64] as well as mapping. Multiple single institution case studies have been presented for radiation segmentectomy and radiation lobectomy in iCCA patients [26, 65–71]. In a study of 10 iCCA patients with 13 tumours undergoing radiation segmentectomy (> 400 Gy) with a median tumour size of 5.3 cm (range, 1.5–13.6 cm), an ORR of 100% by mRECIST was achieved, with one patient each undergoing resection (> 99% necrosis) or transplantation (> 95% necrosis) and with low grade 3 or higher toxicity (6.9%) [70]. The largest case study included 37 procedures in 28 patients [65]; baseline albumin-bilirubin score (ALBI) grades were ALBI 1 in 31/37 (83.8%) and ALBI 2 in 6/37 (16.2%). Segmental (*n* = 18), segmental plus lobar (*n* = 8) and lobar (*n* = 2) infusions were performed. Median tumour size was 6.8 cm (range 2.0–14.0 cm) and 12 (42.9%) patients had multifocal disease. Median perfused volume absorbed dose per patient was 256.8 Gy (range 195.7 – 807.8 Gy). Median perfused volume absorbed dose was 282.9 Gy (range 210.5–807.8 Gy) for segmental infusions, and 328.2 Gy (range 195.7–372.6 Gy) for lobar infusions, which included an ablative segmental combined with a lobar infusion to induce hypertrophy, termed modified radiation lobectomy. Radiation segmentectomy and lobectomy were well tolerated with ≥ grade 3 adverse events occurring in 2 (7.1%) patients. ORR at three months was 94.1%, baseline CA 19–9 decreased significantly and median OS was not reached with a median follow-up of 13.4 months [65]. In a second series of 16 patients (segmental, *n* = 10; lobar, *n* = 6) the average perfused volume absorbed dose was 309.3 ± 199.0 Gy with a median of 225.1 Gy [69]. OS varied and was higher for patients who had radioembolization prior to systemic chemotherapy and in patients who had better liver function (albumin, bilirubin and liver enzyme toxicity grades). Select patients went onto surgical resection or transplant [67, 68, 70]. Central lesions are common in iCCA and radiation segmentectomy has demonstrated applicability [18, 68, 72]. In a third series of 17 patients, a dose response correlation was identified for patients undergoing segment/subsegmental (*n* = 10), 2–3 segments (*n* = 7) or lobar infusions (*n* = 3) [73]. ORR was 94.1% with a mean TAD of 294 ± 0 Gy, 465 ± 292.4 Gy and 951.8 ± 666.5 Gy for SD, PR and complete response (CR), respectively. ROC analysis for CR identified a threshold of > 541.7 Gy with an AUC of 0.738. Patients who achieved a CR had significantly longer OS than patients who did not have a CR, regardless of subsequent therapy (HR = 4.79; *p* = 0.01) [73].

Radioembolization has also been studied in conjunction with first-line systemic chemotherapy [12, 18, 70, 72, 74], as an adjuvant or as consolidation therapy [70, 71, 75, 76] and in chemorefractory patients [70, 71, 77]. Conversion of unresectable patients to resection candidates was commonly reported [12, 18, 72, 74, 76, 78]. In a meta-analysis of 21 studies in 921 iCCA patients, 397 patients were treated with ^90^Y glass radioembolization, of which 11% went onto surgical resection [79]. The meta-analysis noted that higher TAD (median 256 Gy (range, 135–468 Gy) and ≥ 260 Gy) ^90^Y glass radioembolization (alone or combined with surgical resection) improved OS [74, 76]. No clinically significant changes in quality of life were demonstrated in a study that combined systemic chemotherapy with radioembolization [80].

Ablative radiation segmentectomy should increase the number of resection candidates [12, 18, 71, 76]. Ablative dosimetry can easily be achieved with the high specific activity of ^90^Y glass microspheres, even in small treatment volumes [81, 82]. In patients treated with a variety of systemic chemotherapy regimens, a TAD threshold of 260 Gy resulted in a resection rate of 28% [76], and a mean TAD of 322 ± 165 Gy resulted in a conversion to resection rate of 49% [72]. Mean NTAD was not associated with liver toxicity (73.2 ± 25.8 Gy with toxicity and 77.8 ± 16.9 Gy without), only Child–Pugh status and underlying cirrhosis. In a retrospective review of heavily pre-treated patients, a perfused volume absorbed dose of 238.4 ± 130.0 Gy resulted in an ORR of 57.1% by RECIST 1.1, median PFS of 265 days, median OS of 22.9 months, and higher OS in patients undergoing transplant or resection (median not reached; 100% at 1-year and 62.5% at 2-year) [72]. In monotherapy SIRT, radiation lobectomy demonstrated a post-hepatectomy liver failure rate of 3.8% [29].

^90^Y glass microsphere radioembolization was prospectively studied in the first-line setting for 24 treatment-naïve patients using SCD with a median perfused volume absorbed dose of 135.5 Gy [83]. Median hPFS was 5.5 months and median OS was 19.4 months. Patients with solitary tumours and disease control by RECIST 1.1 had prolonged OS. In the MISPHEC phase II study, SIRT monotherapy had fewer grade 3 toxicities (8.0%) versus combination with systemic chemotherapy (71%) [12, 83, 84]; however, fewer patients underwent resection, 4% versus 22% [74, 83].

In the MISPHEC trial, first-line systemic gemcitabine/cisplatin was combined with radioembolization using MCD in unresectable iCCA patients (*n* = 41; target TAD ≥ 205 Gy). After treatment, 22% of patients underwent resection [12]. In most iCCA patients, gemcitabine/cisplatin followed by either modified radiation lobectomy or radiation segmentectomy in combination with capecitabine resulted in conversion to resection (53.8%), and 5/7 downstaged patients had an R0 resection with > 90% necrosis [18]. Cirrhotic iCCA patients undergoing whole liver SIRT experienced high rates of nonreversible hepatic failure (42%) which did not occur in non-cirrhotic patients [74].

Similar to HCC patients, tumour response, tumour biomarker (CA 19-9) and OS are correlated with TAD [72, 76, 77, 79]. In patients treated with ^90^Y glass radioembolization, no dose-toxicity relationship has been established for NTAD [72]. Published values for NTAD are typically below that noted for HCC [12, 72, 74, 76]. Among unresectable iCCA patients, treatment algorithms include selective, lobar, or selective and lobar combined, with the intent of converting to resection [12, 72, 74, 76].

Table 3 lists recommendations for iCCA.

**Table 3:** iCCA radioembolization recommendations

Treatment Intent	Intention may be curative, bridging to curative (transplant or resection), palliation and delay of disease progression, and may be in combination with systemic treatment. Whenever possible, the preferred goal is resection 1. In eligible patients, radiation segmentectomy or radiation lobectomy are recommended 2. Most patients are treated with palliative intent due to late stage, multifocal disease with large tumour load
Patient Selection	1. Longer median OS in patients with ECOG ≤ 1, well-differentiated histology, solitary disease, and absence of extrahepatic disease [75] 2. Patients may have a higher acceptable bilirubin (≤ 1.5 ULN) compared with mCRC and mNET (≤ 1.0 ULN) 3. Maximum acceptable intrahepatic tumour burden is ~ 50% [72]. The higher the tumour burden, the higher the risk for toxicity and/or rapid disease progression (contraindication) 4. Current consensus is to be conservative regarding extrahepatic disease (negative prognostic factor). Studies accepted no or limited extrahepatic disease (e.g., hilar lymph nodes ≤ 3 cm or < 5 lung nodules, each ≤ 10 mm) [12] 5. Patients undergoing more aggressive treatment (radiation lobectomy or higher TAD), should be Child–Pugh A 6. Patients undergoing concomitant systemic chemotherapy as a bridge to resection should be Child–Pugh A5 without underlying cirrhosis [12]. Radioembolization is integrated with chemo-immunotherapy regimens if considered as a first-line therapy 7. Radioembolization may be considered in iCCA with biliary obstruction with percutaneous reconstruction of the biliary hilum
Pretreatment Imaging	1. Arterial and portal venous phase contrast-enhanced MRI or CT is recommended within 4 weeks of treatment to avoid unexpected interval progression 2. [^18^F]FDG-PET/CT (may be combined with CECT) may be performed for staging
Dose Calculation and Dosimetry Considerations	1. For radiation segmentectomy, SCD perfused volume absorbed dose ≥ 400 Gy (no upper limit established) is recommended [65]. MCD and voxel-based dosimetry may become more important in radiation segmentectomy in the future 2. For radiation lobectomy, SCD with a target lobar dose of 140–150 Gy is recommended. For modified radiation lobectomy, a radiation segmentectomy dose of ≥ 400 Gy is recommended with a lobar infusion of 100 Gy [15] 3. MCD is preferred over SCD to evaluate TAD and NTAD. TAD ≥ 205 Gy has been evaluated, providing good response and safety profile [12, 18, 74, 76]. In a retrospective review of 64 patients, OS was higher in patients with a TAD ≥ 260 Gy versus TAD < 260 Gy [76]. A maximum whole liver NTAD of 75 Gy is recommended as a preliminary dose threshold to ensure safety. Proportionally higher NTAD is acceptable for smaller treatment fractions 4. SCD reference average absorbed dose to the perfused volume is 120 Gy. The perfused volume may be the whole liver or a fraction thereof, which has proven to be safe and effective after first-line chemotherapy [12, 72, 77, 85]. Treatment intensification using ≥ 150 Gy to the target liver volume has been evaluated for patients with adequate (≥ 30%) untreated liver reserve [12, 18, 74, 76]. TAD and NTAD may vary considerably between patients. Caution needed in case of poor targeting ([^99m^Tc]TcMAA SPECT/CT) and/or low tumour burden (< 10%), leading to low efficacy and/or high toxicity respectively 5. Dose reductions should be considered in case of toxicity from previous systemic treatment or in case of concomitant systemic treatment, especially radiation-sensitizing treatment
Treatment Delivery	1. Treatment delivery varies based on treatment intent. For curative intent, a selective infusion to ≤ 2 segments is recommended. For bridge to resection, a hepatic reserve of ≥ 30% is recommended 2. For bilobar disease, sequential lobar treatments with an interval of 3–6 weeks is preferred [12]. A shorter interval may decrease the chance of interval progression but may increase cumulative toxicity. A longer interval may increase recovery, but at the potential cost of interval progression (consider repeat imaging) 3. For highly aggressive bilobar disease in a patient with preserved liver function and good [^99m^Tc]TcMAA tumour targeting (i.e., high TAD; low NTAD), single-session bilobar treatment (i.e. two unilobar injections) should be based on MCD
Outcome Assessment and Follow-Up	1. Contrast-enhanced MRI or CT should be performed every 3 months following treatment 2. [^18^F]FDG-PET metabolic response can be seen 4–6 weeks after radioembolization; response at cross-sectional anatomic imaging may take longer (2–3 months) 3. In the palliative setting, caution is warranted with an overly aggressive retreatment approach in patients with SD or PR. Retreatment (radioembolization, ablation, or systemic therapy) should typically be considered only in the setting of PD
Strength of Recommendation	B
Degree of Consensus	Strong

## Discussion

Given the increased use of radioembolization in non-HCC indications, there is an unmet need for treatment and dosimetry guidance. The level of evidence for radioembolization is limited for non-HCC indications, particularly iCCA and mNET, thus, consensus was largely based on the current state of practice, available data, and committee experience. The committee nevertheless notes that data is sufficient to provide recommendations on patient selection, treatment planning, assessment and follow-up. Strong consensus agreement was provided for all discussed indications to support dosimetry-based treatment planning, with any benefits outweighing harms. Although the committee advocates the use of specific dose thresholds for TAD and NTAD, the presented numbers are preliminary and should be used with caution.

Future studies should incorporate personalized dosimetry into study protocols, which includes radiation segmentectomy, radiation lobectomy, MCD and voxel dosimetry, and include both pretreatment and post-treatment dosimetry assessments. Specific areas of focus include: 1) radiation segmentectomy of mCRC, mNET and iCCA patients; 2) bridge to surgery for either resection or transplant oncology where radioembolization is utilized in parallel or in series with existing local or systemic treatment options; 3) comparison of radiation lobectomy to alternative techniques (e.g., portal vein embolization (PVE)) relative to post-hepatectomy liver failure and FLR hypertrophy; 4) pretreatment dosimetry to improve patient selection and treatment planning; 5) publication of patient outcomes based on identified prognostic factors (e.g., mCRC: KRAS status, ECOG status, baseline CEA levels); 6) additional clinical data utilizing radioembolization in parallel or sequence with systemic treatment, i.e., gemcitabine/cisplatin/durvalumab in iCCA, PRRT or gemcitabine/temozolomide in mNET or trifluridine/tipiracil in mCRC; and 7) dosimetry data specific to mCRC, mNET or iCCA to improve radioembolization standardization. In addition, emerging pre-treatment imaging techniques for functional assessment of FLR, such as hepatobiliary scintigraphy, Eovist® (USA), or Primovist® (EU) (gadolinium-EOB-DTPA) MRI may be used more frequently in the future; however, the use of these imaging modalities in routine practice (including thresholds) requires additional studies. Their use in clinical studies is encouraged by the committee, especially in the setting of radiation lobectomy [27, 86].

Although clinical data for mCRC, mNET and iCCA is growing and reports similar dose–response relationships as HCC, more data is needed for treatment optimization and combination with locoregional and systemic treatments. Additional study in indication specific patient selection, treatment planning and combination treatment options will guide improvement in patient outcomes.

## Supplementary Information

Below is the link to the electronic supplementary material.

Supplementary file1 (PDF 230 KB)

## Data Availability

All data reviewed in the creation of these recommendations can be found in the published literature, per the manuscript’s references.
