# Supplementary material for: Clinical and dosimetric considerations for yttrium-90 glass microspheres radioembolization of intrahepatic cholangiocarcinoma, metastatic colorectal carcinoma, and metastatic neuroendocrine carcinoma: recommendations from an international multidisciplinary working group
Source: Eur J Nucl Med Mol Imaging. 2025 Mar 28;52(10):3820–32. doi: 10.1007/s00259-025-07229-8 (PMC12316795; doi:10.1007/s00259-025-07229-8)
Supplement: Supplementary file 1 — Supplementary file1 (PDF 230 KB) [file 259_2025_7229_MOESM1_ESM.pdf]

Clinical and dosimetric considerations for yttrium-90 glass microspheres radioembolization of intrahepatic cholangiocarcinoma, metastatic colorectal carcinoma, and metastatic neuroendocrine carcinoma: Recommendations from an international multidisciplinary working group

Authors: Marnix Lam[1], Riad Salem[2], Beau Toskich[3], S. Cheenu Kappadath[4], Carlo Chiesa[5], Kirk Fowers[6], Paul Haste[7], Joseph M. Herman[8], Edward Kim[9], Thomas Leung[10], Siddharth A Padia[11], Bruno Sangro[12], Daniel Y. Sze[13], Etienne Garin[14]

Affiliations: [1]Department of Radiology and Nuclear Medicine, University Medical Center Utrecht, Utrecht, The Netherlands[1]Univ Rennes, INSERM, INRA, Centre de Lutte contre le Cancer Eugène Marquis, Institut NUMECAN (Nutrition Metabolisms and Cancer), F-35000, Rennes, France; [2]Department of Radiology, Northwestern Feinberg School of Medicine, Chicago, IL, USA; [3] Department of Radiation Oncology, Mayo Clinic, Jacksonville, FL, USA; [4]Department of Imaging Physics, University of Texas MD Anderson Cancer Center, Houston, TX, USA; [5]Department of Nuclear Medicine, Fondazione IRCCS Istituto Nazionale Tumori, Milan, Italy; [6]Boston Scientific Corporation, Marlborough, MA, USA; [7]Department of Radiology and Imaging Sciences, Indiana University School of Medicine, Indianapolis, IN, USA; [8]Department of Radiation Medicine, Northwell Health, New Hyde Park, NY, USA; [9]Department of Interventional Radiology, Mount Sinai, New York City, NY, USA; [10]Comprehensive Oncology Centre, Hong Kong Sanatorium and Hospital, Hong Kong, Hong Kong; [11]Department of Radiology, University of California-Los Angeles, Los Angeles, CA, USA; [12]Liver Unit, Clinica Universidad de Navarra and CIBEREHD, Pamplona, Spain; [13]Department of Radiology, Stanford University School of Medicine, Palo Alto, CA, USA; [14]Department of Nuclear Medicine, Cancer Institute Eugene Marquis, Rennes, France

Online Resources

Online Resource 1: Degree of recommendation

Degree	Meaning
A	Strongly recommended (good evidence that the measure is effective, and benefits outweigh the harms)
B	Recommended (at least moderate evidence that the measure is effective, and benefits exceed harms)
C	No recommendation for or against (at least moderate evidence that the measure is effective, but benefits are similar to harms and a general recommendation cannot be justified)
D	Recommended against (at least moderate evidence that the measure is ineffective or that the harms exceed the benefits)
E	Insufficient, low quality or contradictory evidence (the balance between benefit and harms cannot be determined)

Online Resource 2: Strength of consensus

Strength of consensus	% consensus
Strong	≥80
Moderate	50–79
Weak	≤49

Online Resource 3: Methods

Dosimetry planning for yttrium-90 (^{90}Y) glass microspheres is proposed with a mean dose approach, i.e., aiming for an average dose over either single or multicompartment volumes of interests (VOIs). Published data is limited on voxel-based dosimetry (non-uniform distribution), therefore voxel-based dosimetry was not included in the current recommendations [1].

Technetium-99 macroaggregated albumin ([^{99m}Tc]TcMAA) is administered during the mapping angiogram to assess lung shunting, potential extrahepatic non-target deposition through hepatico-enteric collateral vessels and intrahepatic multicompartment distribution of activity. [^{99m}Tc]TcMAA tumour uptake may be variable across tumour types. Typically, neuroendocrine tumours (NET) has a high tumour uptake, metastatic colorectal cancer (mCRC) has a low tumour uptake while intrahepatic cholangiocarcinoma

(iCCA) may have variable tumour uptake [2]. The scout dose [^{99m}Tc]TcMAA may be injected in the proper hepatic artery, selectively in both lobes, or selectively in one lobe (highest burden). However, it is important to recognize that in order to implement [^{99m}Tc]TcMAA for intrahepatic dosimetry (multicompartment dosimetry; MCD), injection of [^{99m}Tc]TcMAA and ^{90}Y glass microspheres should be performed at the same angiographic position, minimizing the influence of vessel bifurcations, i.e., preferential flow, and injected slowly (20–30 seconds), in order to mimic microsphere infusion [3]. It is important to note that [^{99m}Tc]TcMAA overestimates lung shunting. This overestimation is large and systematic especially when planar imaging is used [1]. SPECT/CT provides more accurate lung shunting assessment, but differences in MAA particle size and free [^{99m}Tc]TcPertechnetate will result in overestimation [1]. According to SPECT/CT evaluation, lower dose threshold should be used (<30 Gy planar, <20 Gy SPECT/CT) [4-7]. Contemporary practice also supports the use of post-therapy imaging with ^{90}Y bremsstrahlung SPECT/CT or ^{90}Y PET/CT with the intent to (1) verify dose delivery and distribution, (2) gain information that facilitates calculation of normal tissue absorbed dose (NTAD) and tumour absorbed dose (TAD), thereby assisting with expectation of clinical response or complications, and (3) have information that can be used as an input for retreatment dosimetry [7].

Key definitions used throughout this document are identical to those in the hepatocellular carcinoma (HCC)-related recommendations [8, 9]:

- **Mean absorbed dose:** quantity expressed in Gray (Gy) to describe the average amount of energy (joules) deposited within a specific VOI with a given mass (kg). The mean absorbed dose is often referred to as "Dose" and must not be mistaken for "Activity" or "Dosage" (GBq) [10, 11].

- **MIRD schema:** The Medical Internal Radiation Dose (MIRD) schema is applicable to both the single compartment dosimetry (SCD) and MCD models. The mean absorbed dose (*D*) in any specific VOI (i.e., perfused volume, lobe, tumour or normal tissue) with mass of any VOI, denoted as *M*, with the assumption that *D* is distributed uniformly in each volume with permanent

microsphere implantation and no biological clearance [12, 13]. Using this schema, D in a VOI is computed as:

$$D_{(\mathrm{Gy})} = \frac{A_{(\mathrm{GBq})} \times (50_{(\mathrm{Gy\ x\ kg/GBq})}(1 - F)}{M_{(\mathrm{kg})}}$$

where A is the net activity of ^{90}Y in the VOI, and F is the lung shunt fraction [14, 15].

- **Single compartment model:** In the single compartment model, only the averaged absorbed dose value over the treated liver volume can be calculated, neglecting the difference in microsphere distribution patterns (especially between tumours and normal liver). This represents a simplification of the real distribution. Hypervascular tumours will receive a higher dose, and the normal parenchyma will receive a lower absorbed dose [16-19].
- **Multicompartment model:** A dosimetry model, based on the MIRD schema, where D is determined in more than one VOI, such as the tumour VOI and the normal parenchyma VOI. Partition modelling refers to the MCD approach reporting the tumoural and non-tumoural doses separately with a single averaged tumour to averaged non-tumoural uptake ratio (T:N ratio) [15, 19, 20].

Online Resource 4: Recommendations for mCRC, mNET and iCCA

**Treatment Intent**	1. Liver function should be preserved, and radioembolization-induced liver disease (REILD) and late onset liver toxicities including liver fibrosis should be prevented, especially in situations where expected survival is long enough that subsequent treatment is possible (e.g., surgery in the setting of radiation lobectomy, repeat radioembolization, local ablative therapies, or systemic therapy). For this purpose, except in case of superselective treatment, it is advocated to use multicompartment dosimetry and plan whole normal liver dose below safety limits, considering risk factors (baseline liver function, lobar therapy in patients with portal hypertension, previous chemotherapy lines, or other hepatotoxic treatments).<br>2. Exceptions to palliative intent treatment include radiation segmentectomy with the intention of complete tumour ablation (i.e., treatment of up to two Couinaud

	segment(s) or up to 25% whole liver volume) [21], and radiation lobectomy with the aim to control ipsilateral disease and induce contralateral hypertrophy in preparation for potential resection. Ablative or bridge to resection intent have implications for treatment planning and dosimetry (see below). Since major portions of the liver remain untreated in these situations (i.e., safety assurance), treatment is geared towards maximal efficacy of the treated volume.<br>3. The intent of radiation segmentectomy is complete tumour ablation [22, 23]. At these high ablative absorbed dose levels, treatment effect relies less on tumour type and will be effective in non-HCC tumours [13, 24-27].<br>4. Radiation lobectomy applies to patients who are potential resection candidates, but: a) have inadequate future liver remnant (FLR), and/or b) need an embedded test-of-time to better define tumour biology, and/or c) need the treated tumour to be retracted from the hepatic vein and/or inferior vena cava, and/or d) need demonstration of a tumour response prior to surgery [28, 29].
**Patient Selection**	1. Life expectancy of ≥3 months, ECOG score 0-1 [24, 30, 31].<br>2. Patients should have sufficient liver function (i.e., aspartate aminotransferase or alanine aminotransferase <5 times upper limit of normal (ULN), INR ≤1.5, albumin ≥3.0 g/dL and normal bilirubin). Treatment is contraindicated when bilirubin exceeds normal levels and is not caused by biliary obstruction, especially in bilobar metastatic disease, where whole liver treatment is warranted [31, 32]. In case of more severe hepatic dysfunction, consider multidisciplinary discussion on individualized patient characteristics and consider other treatment options.<br>3. Sufficient renal function to allow for angiography (i.e., creatinine clearance >30 ml/min). No data in patients on dialysis is available.<br>4. Pretreatment [^{99m}Tc]TcMAA distribution may be part of patient selection. In case of grossly inadequate TAD and/or overtly unacceptable NTAD, patients should not be treated because of futility and/or safety concerns, respectively. It should be noted: 1) [^{99m}Tc]TcMAA may not always be a reliable predictor of post-treatment distribution, 2) specific dose thresholds are preliminary (see below), and 3) pretreatment CT/MRI alone is insufficient to determine vascularity/suitability.<br>5. Tumours abutting the colon, gallbladder and stomach can usually be safely treated in the absence of prior resection or adjuvant, high-dose radiation, which may cause adherence of these structures and potential collateral damage. Extrahepatic radiation toxicity to adjacent structures has been demonstrated in only a few case reports [33-35]. In the setting of biliary stents post-sphincterotomy, administration of prophylactic antibiotics is recommended due to risk of abscess formation [36].
**Treatment Planning**	Multiple variations of [^{99m}Tc]TcMAA administration exist. Options include [9]:<br>1. Inject [^{99m}Tc]TcMAA in the proper hepatic artery to perfuse the entire liver. This approach is sufficient for lung shunt fraction estimation, but may not be accurate for dosimetry purposes in case treatment is delivered in a selective approach (i.e., not in the proper hepatic artery, but in the right and left hepatic artery selectively).

	2. Injection in the lobe with higher tumour burden. This option is used for superselective approaches to limit the number of catheterizations of the small segmental branch, perfusing tumour [37]. This approach is sufficient for lung shunt fraction estimation (most conservative), and the contralateral lobe, but may not be accurate for dosimetry purposes.<br>3. Injection in both lobes with a split vial of [^{99m}Tc]TcMAA into right and left hepatic arteries (ideal for MCD) [38]. To optimize MCD treatment planning the catheter position for [^{99m}Tc]TcMAA and TheraSphere should be consistent [39].<br>4. The nominal [^{99m}Tc]TcMAA activity is 150 MBq (e.g., 100 MBq injected in the right lobe; 50 MBq injected in the left lobe). Note: the interval between preparation and administration does not influence MAA distribution, but the interval between administration and imaging does. Biodegradation, leading to higher extrahepatic activity (e.g., lung shunt fraction, thyroid, kidneys and stomach) starts immediately after administration [39].<br>5. Acceptable lung absorbed dose is 30 Gy for a single treatment and 50 Gy cumulative for multiple treatments, based on planar [^{99m}Tc]TcMAA scintigraphy. However, dose-effect relationships have not been established (because of low incidence of radiation-induced pneumonitis, <1 %). Furthermore, It is generally recognized that 1) [^{99m}Tc]TcMAA in SPECT/CT largely overestimates the lung shunt fraction, 2) [^{99m}Tc]TcMAA quantification on planar scintigraphy largely overestimates the lung activity (in part because of lack of attenuation correction), and 3) [^{99m}Tc]TcMAA lung shunt fraction increases at longer intervals between administration and imaging.<br><br>Intra-procedural CT (cone-beam CT or Angio-CT)<br>It is highly recommended to use a cross-sectional technique during the mapping procedure:<br>1. General: to identify extrahepatic perfusion from hepatico-enteric vessels and to determine the perfused volume(s).<br>2. Radiation segmentectomy: to determine angiosome volume by cone-beam CT or angio-CT [22, 23].
**Strength of Recommendation**	B
**Degree of Consensus**	Strong

Online Resource References

1. Chiesa C, Sjogreen-Gleisner K, Walrand S, Strigari L, Flux G, Gear J, et al. EANM dosimetry committee series on standard operational procedures: a unified methodology for (99m)Tc-MAA pre- and (90)Y peri-therapy dosimetry in liver radioembolization with (90)Y microspheres. EJNMMI Phys. 2021;8(1):77. DOI:10.1186/s40658-021-00394-3.

2. Ilhan H, Goritschan AM, Paprottka P, Jakobs TF, Fendler WP, Bartenstein P, et al. Systematic Evaluation of Tumoral 99mTc-MAA Uptake Using SPECT and SPECT/CT in 502 Patients Before 90Y Radioembolization. The Journal of Nuclear Medicine. 2015;56:333 - 8.

3. Garin E, Rolland Y, Laffont S, Edeline J. Clinical impact of (99m)Tc-MAA SPECT/CT-based dosimetry in the radioembolization of liver malignancies with (90)Y-loaded microspheres. Eur J Nucl Med Mol Imaging. 2016;43(3):559-75. DOI:10.1007/s00259-015-3157-8.

4. Lopez B, Mahvash A, Lam M, Kappadath SC. Calculation of lung mean dose and quantification of error for (90) Y-microsphere radioembolization using (99m) Tc-MAA SPECT/CT and diagnostic chest CT. Med Phys. 2019;46(9):3929-40. DOI:10.1002/mp.13575.

5. Kappadath SC, Henry EC, Lopez BP, Mahvash A. Quantitative evaluation of (90)Y-PET/CT and (90)Y-SPECT/CT-based dosimetry following Yttrium-90 radioembolization. Med Phys. 2024;51(9):6061-74. DOI:10.1002/mp.17175.

6. Allred JD, Niedbala J, Mikell JK, Owen D, Frey KA, Dewaraja YK. The value of (99m)Tc-MAA SPECT/CT for lung shunt estimation in (90)Y radioembolization: a phantom and patient study. EJNMMI Res. 2018;8(1):50. DOI:10.1186/s13550-018-0402-8.

7. Chan KT, Alessio AM, Johnson GE, Vaidya S, Kwan SW, Monsky W, et al. Prospective Trial Using Internal Pair-Production Positron Emission Tomography to Establish the Yttrium-90 Radioembolization Dose Required for Response of Hepatocellular Carcinoma. Int J Radiat Oncol Biol Phys. 2018;101(2):358-65. DOI:10.1016/j.ijrobp.2018.01.116.

8. Salem R, Padia SA, Lam M, Bell J, Chiesa C, Fowers K, et al. Clinical and dosimetric considerations for Y90: recommendations from an international multidisciplinary working group. Eur J Nucl Med Mol Imaging. 2019;46(8):1695-704. DOI:10.1007/s00259-019-04340-5.

9. Salem R, Padia SA, Lam M, Chiesa C, Haste P, Sangro B, et al. Clinical, dosimetric, and reporting considerations for Y-90 glass microspheres in hepatocellular carcinoma: updated 2022 recommendations from an international multidisciplinary working group. Eur J Nucl Med Mol Imaging. 2023;50(2):328-43. DOI:10.1007/s00259-022-05956-w.

10. Bolch WE, Eckerman KF, Sgouros G, Thomas SR. MIRD pamphlet No. 21: a generalized schema for radiopharmaceutical dosimetry--standardization of nomenclature. J Nucl Med. 2009;50(3):477-84. DOI:10.2967/jnumed.108.056036.

11. The International Commission on Radiation Units and Measurements. Journal of the ICRU. 2011;11(1):5-6. DOI:10.1093/jicru_ndr011.

12. Ebbers SC, van Roekel C, Braat M, Barentsz MW, Lam M, Braat A. Dose-response relationship after yttrium-90-radioembolization with glass microspheres in patients with neuroendocrine tumor liver metastases. Eur J Nucl Med Mol Imaging. 2022;49(5):1700-10. DOI:10.1007/s00259-021-05642-3.

13. Padia SA, Johnson GE, Agopian VG, DiNorcia J, Srinivasa RN, Sayre J, et al. Yttrium-90 radiation segmentectomy for hepatic metastases: A multi-institutional study of safety and efficacy. J Surg Oncol. 2021;123(1):172-8. DOI:10.1002/jso.26223.

14. Dezarn WA, Cessna JT, DeWerd LA, Feng W, Gates VL, Halama J, et al. Recommendations of the American Association of Physicists in Medicine on dosimetry, imaging, and quality assurance procedures for 90Y microsphere brachytherapy in the treatment of hepatic malignancies. Med Phys. 2011;38(8):4824-45. DOI:10.1118/1.3608909.

15. Gulec SA, Mesoloras G, Stabin M. Dosimetric techniques in 90Y-microsphere therapy of liver cancer: The MIRD equations for dose calculations. J Nucl Med. 2006;47(7):1209-11.

16. Salem R, Thurston KG. Radioembolization with 90Yttrium microspheres: a state-of-the-art brachytherapy treatment for primary and secondary liver malignancies. Part 1: Technical and methodologic considerations. J Vasc Interv Radiol. 2006;17(8):1251-78. DOI:10.1097/01.RVI.0000233785.75257.9A.

17. Salem R, Thurston KG. Radioembolization with 90yttrium microspheres: a state-of-the-art brachytherapy treatment for primary and secondary liver malignancies. Part 2: special topics. J Vasc Interv Radiol. 2006;17(9):1425-39. DOI:10.1097/01.RVI.0000235779.88652.53.

18. Salem R, Thurston KG, Carr BI, Goin JE, Geschwind JF. Yttrium-90 microspheres: radiation therapy for unresectable liver cancer. J Vasc Interv Radiol. 2002;13(9 Pt 2):S223-9. DOI:10.1016/s1051-0443(07)61790-4.

19. Mikell JK, Mahvash A, Siman W, Baladandayuthapani V, Mourtada F, Kappadath SC. Selective Internal Radiation Therapy With Yttrium-90 Glass Microspheres: Biases and Uncertainties in Absorbed Dose Calculations Between Clinical Dosimetry Models. Int J Radiat Oncol Biol Phys. 2016;96(4):888-96. DOI:10.1016/j.ijrobp.2016.07.021.

20. Ho S, Lau WY, Leung TW, Chan M, Ngar YK, Johnson PJ, et al. Partition model for estimating radiation doses from yttrium-90 microspheres in treating hepatic tumours. Eur J Nucl Med. 1996;23(8):947-52. DOI:10.1007/bf01084369.

21. De la Garza-Ramos C, Overfield CJ, Montazeri SA, Liou H, Paz-Fumagalli R, Frey GT, et al. Biochemical Safety of Ablative Yttrium-90 Radioembolization for Hepatocellular Carcinoma as a Function of Percent Liver Treated. J Hepatocell Carcinoma. 2021;8:861-70. DOI:10.2147/JHC.S319215.

22. Salem R, Johnson GE, Kim E, Riaz A, Bishay V, Boucher E, et al. Yttrium-90 Radioembolization for the Treatment of Solitary, Unresectable HCC: The LEGACY Study. Hepatology. 2021;74(5):2342-52. DOI:10.1002/hep.31819.

23. Kim E, Sher A, Abboud G, Schwartz M, Facciuto M, Tabrizian P, et al. Radiation segmentectomy for curative intent of unresectable very early to early stage hepatocellular carcinoma (RASER): a single-centre, single-arm study. Lancet Gastroenterol Hepatol. 2022;7(9):843-50. DOI:10.1016/S2468-1253(22)00091-7.

24. Ahmed O, Yu Q, Patel M, Hwang G, Pillai A, Liao CY, et al. Yttrium-90 Radioembolization and Concomitant Systemic Gemcitabine, Cisplatin, and Capecitabine as the First-Line Therapy for Locally Advanced Intrahepatic Cholangiocarcinoma. J Vasc Interv Radiol. 2023;34(4):702-9. DOI:10.1016/j.jvir.2022.12.017.

25. Meiers C, Taylor A, Geller B, Toskich B. Safety and initial efficacy of radiation segmentectomy for the treatment of hepatic metastases. J Gastrointest Oncol. 2018;9(2):311-5. DOI:10.21037/jgo.2017.11.02.

26. Kurilova, Bendet, Fung, Petre, Humm, Boas, et al. Radiation segmentectomy of hepatic metastases with Y-90 glass microspheres. Abdominal Radiology. 2021;46:3428 - 36.

27. Chiu AM, Savoor R, Gordon AC, Riaz A, Sato KT, Hohlastos E, et al. Yttrium-90 Radiation Segmentectomy in Oligometastatic Secondary Hepatic Malignancies. Journal of Vascular and Interventional Radiology. 2023;34(3):362-8. DOI:10.1016/j.jvir.2022.12.021.

28. Vouche M, Lewandowski RJ, Atassi R, Memon K, Gates VL, Ryu RK, et al. Radiation lobectomy: time-dependent analysis of future liver remnant volume in unresectable liver cancer as a bridge to resection. J Hepatol. 2013;59(5):1029-36. DOI:10.1016/j.jhep.2013.06.015.

29. Shah JL, Zendejas-Ruiz IR, Thornton LM, Geller BS, Grajo JR, Collinsworth A, et al. Neoadjuvant transarterial radiation lobectomy for colorectal hepatic metastases: a small cohort analysis on safety, efficacy, and radiopathologic correlation. J Gastrointest Oncol. 2017;8(3):E43-e51. DOI:10.21037/jgo.2017.01.26.

30. Edeline J, Touchefeu Y, Guiu B, Farge O, Tougeron D, Baumgaertner I, et al. Radioembolization Plus Chemotherapy for First-line Treatment of Locally Advanced Intrahepatic Cholangiocarcinoma: A Phase 2 Clinical Trial. JAMA Oncol. 2020;6(1):51-9. DOI:10.1001/jamaoncol.2019.3702.

31. Mulcahy MF, Mahvash A, Pracht M, Montazeri AH, Bandula S, Martin RCG, 2nd, et al. Radioembolization With Chemotherapy for Colorectal Liver Metastases: A Randomized, Open-Label, International, Multicenter, Phase III Trial. J Clin Oncol. 2021;39(35):3897-907. DOI:10.1200/JCO.21.01839.

32. Hickey R, Lewandowski RJ, Prudhomme T, Ehrenwald E, Baigorri B, Critchfield J, et al. 90Y Radioembolization of Colorectal Hepatic Metastases Using Glass Microspheres: Safety and Survival Outcomes from a 531-Patient Multicenter Study. J Nucl Med. 2016;57(5):665-71. DOI:10.2967/jnumed.115.166082.

33. Zhou W, Sze DY. Gastric Outlet Obstruction Following Radioembolization: Extrahepatic Complication from Proximity to a Superficial Hepatic Tumor Treated with an Ablative Dose. J Vasc Interv Radiol. 2021;32(12):1699-701. DOI:10.1016/j.jvir.2021.09.001.

34. Jung JW, Foster HS, Tabori NE, Sivananthan G. Yttrium-90 Radioembolization Complicated by Colitis. J Vasc Interv Radiol. 2021;32(12):1698-9. DOI:10.1016/j.jvir.2021.07.016.

35. Laidlaw GL, Johnson GE. Recognizing and Managing Adverse Events in Y-90 Radioembolization. Semin Intervent Radiol. 2021;38(4):453-9. DOI:10.1055/s-0041-1735617.

36. Devulapalli KK, Fidelman N, Soulen MC, Miller M, Johnson MS, Addo E, et al. (90)Y Radioembolization for Hepatic Malignancy in Patients with Previous Biliary Intervention: Multicenter Analysis of Hepatobiliary Infections. Radiology. 2018;288(3):774-81. DOI:10.1148/radiol.2018170962.

37. Kallini JR, Gabr A, Kulik L, Salem R, Lewandowski RJ. The Utility of Unilobar Technetium-99m Macroaggregated Albumin to Predict Pulmonary Toxicity In Bilobar Hepatocellular Carcinoma prior to Yttrium-90 Radioembolization. J Vasc Interv Radiol. 2016;27(9):1453-6. DOI:10.1016/j.jvir.2016.06.004.

38. Thomas MA, Mahvash A, Abdelsalam M, Kaseb AO, Kappadath SC. Planning dosimetry for (90) Y radioembolization with glass microspheres: Evaluating the fidelity of (99m) Tc-MAA and partition model predictions. Med Phys. 2020;47(10):5333-42. DOI:10.1002/mp.14452.

39. Weber M, Lam M, Chiesa C, Konijnenberg M, Cremonesi M, Flamen P, et al. EANM procedure guideline for the treatment of liver cancer and liver metastases with intra-arterial radioactive compounds. Eur J Nucl Med Mol Imaging. 2022;49(5):1682-99. DOI:10.1007/s00259-021-05600-z.
